# Supplementary material for: Improving recognition of common mental health disorders in Cambodia: Validation of the PHQ-9 and GAD-7 and development of a brief mental health screener
Source: PLOS Ment Health. 2025 Apr 10;2(4):e0000228. doi: 10.1371/journal.pmen.0000228 (PMC12798203; doi:10.1371/journal.pmen.0000228)
Supplement: S1 Appendix — (DOCX) [file pmen.0000228.s001.docx]

**S1 Appendix. Study measures**

**Hopkins Symptom Checklist (HSCL-25)**

| **Item in English** | **Item in Khmer** |
| --- | --- |
| **Please carefully decide how much these things bothered you in the PAST WEEK……** | ***សូមលោកអ្នក ធ្វើការគិត និងសម្រេចចិត្តអោយបានល្អិតល្អន់ តើអាការៈដូចខាងក្រោមនេះ រំខានអ្នកកំរិតណាកាលពី១សប្តាហ៍កន្លងទៅនេះ…….*** |
| *0 "Not at all", 1 "A little", 2 "Quite a bit", 3 "Extremely"* | *០ "មិនមានទាល់តែសោះ", ១ "មានបន្តិចបន្តួច", ២ "មានច្រើនគួរសម", ​​​​​​​​​​​​​​​​​​​​​​​​​​​​​​​​​​​ ៣ "មានខ្លាំងបំផុត"* |
| Feeling low in energy, slowed down. | មានអារម្មណ៌ខ្សោយកំលាំង ឬស្ទក់ |
| Blaming yourself for things. | ស្តីបន្ទោសខ្លួនឯងអំពីរឿងអ្វីដែលកើតមានឡើង |
| Crying easily. | ងាយឆាប់យំ |
| Loss of sexual interest or pleasure. | ​បាត់បង់នូវចំនង់ផ្លូវភេទ ឬអស់តម្រេកផ្លូវភេទ​ |
| Poor appetite. | មិនសូវឃ្លាន ញាំមិនសូវបាន |
| Difficulty falling asleep, staying asleep. | ពិបាកគេងលក់ គេងលក់ពុំបានយូរ |
| Feeling hopeless about the future. | មានអារម្មណ៌អស់សង្ឃឺមអំពីអនាគត |
| Feeling sad. | មានអារម្មណ៌ក្រៀមក្រំ |
| Feeling lonely. | មានអារម្មណ៍ឯកោ |
| Thoughts of ending your life. | មានគំនិតក្នុងការបញ្ចប់ជីវិតរបស់អ្នក​ |
| Feeling of being trapped or caught. | មានអារម្មណ៍ដូចជាប់អន្ទាក់ដោយសារបញ្ហារបស់ខ្លួន |
| Worrying too much about things. | ព្រួយបារម្ភខ្លាំងពេកអំពីអ្វីៗដែលបានកើតឡើង |
| Feeling no interest in things. | លែងមានចំណាប់អារម្មណ៍លើអ្វីៗទាំងអស់ |
| Feeling everything is an effort. | មានអារម្មណ៌ថា អ្វីៗក៏ពិបាកទាំងអស់​ |
| Feelings of worthlessness. | ​មានអារម្មណ៌ខ្លួនឯងគ្មានតម្លៃ​ |
| ​Faintness, dizziness, or weakness. | ងងឹតមុខ វិលមុខ ឬខ្សោយកំលាំង |
| Feeling fearful. | ​ មានអារម្មណ៌ភ័យខ្លាច​ |
| Feeling restless, can’t sit still. | មានអារម្មណ៍រសាប់រសល់នៅមិនស្ងៀម |
| Feeling tense or keyed up. | ​ មានអារម្មណ៌តឹងតែង ឬរំជើបរំជួល​ |
| Headaches. | មានអារម្មណ៍ឈឺក្បាលខ្លាំង |
| Heart pounding or racing. | បេះដូងលោតខ្លាំង ឬដើរញាប់ |
| Nervousness or shakiness inside. | ជ្រួលច្រាល ឬញ័រក្នុងខ្លួន |
| Spells of terror or panic. | មានអារម្មណ៍ថា ភ័យខ្លាំង ឬស្លន់ស្លោរ |
| Suddenly scared for no reason. | ភ័យខ្លាចមួយរំពេចដោយគ្មានហេតុផល |
| Trembling. | ​ ​ញាប់ញ័រខ្លួនប្រាណ ​ |

**Harvard Trauma Questionnaire (HTQ)**

| **Item in English** | **Item in Khmer** |
| --- | --- |
| **Please carefully decide how much these things bothered you in the PAST WEEK?** | **សូមលោកអ្នក ធ្វើការគិត និងសម្រេចចិត្តអោយបានល្អិតល្អន់ តើអាការៈដូចខាងក្រោមនេះ រំខានអ្នកកំរិតណា កាលពី​សប្តាហ៌មុន…….** |
| *0 "Not at all", 1 "A little", 2 "Quite a bit", 3 "Extremely"* | *០ "មិនមានទាល់តែសោះ", ១ "មានបន្តិចបន្តួច", ២ "មានច្រើនគួរសម", ​​​​​​​​​​​​​​​​​​​​​​​​​​​​​​​​​​​ ៣ "មានខ្លាំងបំផុត"* |
| Recurring thoughts or memories of the most hurtful or terrifying events. | ចេះគិតឬនឹកឃើញជារឿយៗទៅដល់ព្រឹត្តិការណ៍ដ៏ឈឺចាប់ ឬគួរឱ្យតក់ស្លុត |
| Feeling that you have no one to rely on. | ​មានអារម្មណ៌ថា គ្មាននរណាម្នាក់ជាទីពឹង |
| Feeling as though the hurtful or terrifying event is happening again. | ​មានអារម្មណ៍ថា ហាក់ដូចជាហេតុការណ៍ដែលឈឺចាប់និងគួរឱ្យតក់ស្លុតកំពុងកើតឡើងម្តងទៀត |
| Finding out or being told by other people that you have done something that you cannot remember. | នៅពេលដែលអ្នកដឹងថា ឬមាននរណាម្នាក់ប្រាប់ថា អ្នកបានធ្វើអ្វីមួយ ដែលអ្នកមិនអាចចងចាំ |
| Recurrent nightmares. | ​យល់សប្តិអាក្រក់ឡើងវិញដដែលៗ |
| Feeling as if you are split into two people and one of you is watching what the other is doing. | ​មានអារម្មណ៍ហាក់ដូចជា ខ្លួនអ្នកត្រូវបែងចែកជា២ ម្នាក់ចាំមើលនូវអ្វីដែលម្នាក់ទៀតកំពុងធ្វើ |
| Feeling detached or withdrawn from people. | ​មានអារម្មណ៏ថាខ្លួននៅឆ្ងាយ រឺកាត់ផ្តាច់ពីអ្នកដទៃ |
| Feeling someone you trusted betrayed you. | ​មានអារម្មណ៌ថា មាននរណាម្នាក់ដែលអ្នកទុកចិត្ត បានក្បត់អ្នក |
| Unable to feel emotions. | ​ដឹងថា ខ្លួនឯងមិនមានមនោសញ្ចេតនា ឬមិនមានអារម្មណ៌អ្វីទាំងអស់ |
| Feeling jumpy or easily startled. | ​មានអារម្មណ៌ថា ភ័យផ្អើលៗ |
| Difficulty concentrating. | ​មានអារម្មណ៌ថា ពិបាកក្នុងការផ្ចង់ អារម្មណ៌ |
| Trouble sleeping. | ​មានអារម្មណ៌ថា ពិបាកក្នុងការគេង |
| Feeling on guard. | មានអារម្មណ៌ថា ប្រយត្ន័ប្រយែងខ្ពស់ |
| Feeling irritable or having outburst of anger. | ​មានអារម្មណ៌ថាឆាប់ខឹង ឬ ងាយផ្ទុះកំហឹង |
| Avoiding activities that remind you of the traumatic or hurtful event. | ​ព្យាយាមគេចចេញពីសកម្មភាពណា ដែលធ្វើអោយអ្នកនឹកឃើញ ហេតុការណ៌ប៉ះដង្គិចផ្លូវចិត្ត ឬឈឺចាប់ |
| Inability to remember parts of the most traumatic or hurtful events. | ​មិនអាច ចងចាំឬ នឹកឃើញ នូវផ្នែកខ្លះនៃហេតុការណ៌ប៉ះដង្គិចផ្លូវចិត្ត ឬហេតុការណ៍ដែលធ្វើអោយអ្នកឈឺចាប់បំផុត |
| Less interest in daily activities. | មិនសូវចាប់អារម្មណ៍នឹងសកម្មភាពប្រចាំថ្ងៃ |
| Feeling as if you don't have a future. | មានអារម្មណ៍ ហាក់ដូចជាខ្លួនមិនមានអនាគត |
| Avoiding thoughts or feelings associated with the traumatic or hurtful events. | ​ព្យាយាមគេចវេស ការគិត ឬ អារម្មណ៌ទាំងឡាយណាដែលពាក់ពន្ធ័នឹងហេតុការណ៌ប៉ះដង្គិចផ្លូវចិត្ត ឬឈឺចាប់ |
| Sudden emotional or physical reaction when reminded of the most hurtful or traumatic events. | ឆាប់មានប្រតិកម្មរាងកាយ ឬផ្លូវចិត្តភ្លាមៗ នៅពេលដែលគេធ្វើអោយនឹកឃើញដល់ហេតុការណ៍ដែលធ្វើអោយឈឺចាប់បំផុត ឬហេតុការណ៍នៃការប៉ះដង្កិចផ្លូវចិត្ត |
| Feeling that people do not understand what happened to you. | ​មានអារម្មណ៌ថា អ្នកដទៃមិនយល់អំពីអ្វីដែលបានកើតឡើង ចំពោះអ្នក |
| Difficulty performing work or daily tasks. | ​មានការពិបាក ក្នុងការបំពេញការងារប្រចាំថ្ងៃរបស់អ្នក |

**Patient Health Questionnaire (PHQ-9)**

| **Item​ in English** | **Item in Khmer** |
| --- | --- |
| **Over the PAST 2 WEEKS, how often have you been bothered by…​​​** | **តើញឹកញាប់កំរិតណា ដែលអ្នកត្រូវបានរំខានដោយ អាការៈខាងក្រោម៖** |
| *0 "not at all", 1 "several days", 2 "more than half the days of the 2 week period?" , 3 "nearly every day"* | *០ "មិនមានទាល់តែសោះ", ១ "ពីរបីថ្ងៃ", ២ "ច្រើនជាងមួយសប្តាហ៌",​​​​​​​​​​​​​​​​​​​​​​​​​​​​​​​​ ៣ "មានស្ទើរតែគ្រប់ពេល ឬរាល់ថ្ងៃ"* |
| Little interest or pleasure in doing things | មានការចាប់អារម្មណ៍តិចតួចឬសប្បាយចិត្ត តិចតួចក្នុងការធ្វើការងារ ផ្សេងៗ |
| Feeling down, depressed, or hopeless | មានអារម្មណ៍ ស្រងូតស្រងាត់ ធ្លាក់ទឹកចិត្ត​ ឬអស់សង្ឃឹម… |
| ​Trouble falling, staying asleep, or sleeping too much | ពិបាកក្នុងការគេង ឬគេងលង់លក់ពិបាកងើប ឬគេងច្រើនម៉ោងពេក |
| ​Feeling tired or having little energy | មានអារម្មណ៍អស់កម្លាំង ឬ មានកម្លាំងពលំតិចតួច |
| Poor appetite or overeating | មិនចង់បរិភោគអាហារ ឬ ពិសារអាហារច្រើនពេក |
| Feeling bad about oneself or that you are a failure or make yourself or down your family | មានអារម្មណ៍មិនល្អចំពោះខ្លួនឯង ឬមានអារម្មណ៍ថាខ្លួនមិនជោគជ័យ ធ្វើឲ្យខ្លួនឯង ឬគ្រួសាររបស់ខ្លួនអាប់អោនកិត្តិយស.... |
| Trouble concentrating on things, such as reading the newspaper or watching television | ពិបាកក្នុងការប្រមូលអារម្មណ៍ទៅលើអ្វីមួយ ដូចជាការអានកាសែត ឬមើលទូរទស្សន៍…. |
| Moving or speaking so slowly that other people could have noticed? Or the opposite ​being so fidgety or restless that​​​​​ you have been moving around a lot more than usual.. | ប្រើចលនាកំរើក ឬ និយាយយឺតពេកដែល ធ្វើឲ្យមនុស្សឯទៀតកត់សម្គាល់បាន? ឬ ផ្ទុយទៅវិញ រពិសពេក ឬនៅមិនស្ងៀមដែលទៅនេះទៅនោះ ច្រើនជាងធម្មតា |
| Thoughts that you would be better off dead​ or of hurting yourself in some​ways… | មានគំនិតថា បើអ្នកស្លាប់ទៅប្រសើរជាងអ្នករស់នៅ ឬចង់តែធ្វើបាបខ្លួនឯង ដោយមធ្យោបាយផ្សេងៗ |

**Generalized Anxiety Disorder scale (GAD-7)**

| **Item​ in English** | **Item in Khmer** |
| --- | --- |
| **Over the PAST 2 WEEKS, how often have you been bothered by…​​​** | **តើញឹកញាប់កំរិតណា ដែលអ្នកត្រូវបានរំខានដោយ អាការៈខាងក្រោម៖** |
| *0 "not at all", 1 "several days", 2 "more than half the days of the 2 week period?" , 3 "nearly every day"* | *០ "មិនមានទាល់តែសោះ", ១ "ពីរបីថ្ងៃ", ២ "ច្រើនជាងមួយសប្តាហ៌",​​​​​​​​​​​​​​​​​​​​​​​​​​​​​​​​ ៣ "មានស្ទើរតែគ្រប់ពេល ឬរាល់ថ្ងៃ"* |
| Feeling nervous or on edge | មានអារម្មណ៌ច្រួលច្រាលក្នុងចិត្ត​ អន្ទះអន្ទែង ឬតានតឹងក្នុងចិត្ត |
| Not being able to stop or control worrying | មិនអាចបញ្ឈប់ ឬគ្រប់គ្រងការព្រួយបារម្ភបាន |
| Worrying too much about different things | កង្វល់ច្រើនពេកពីរឿងផ្សេងៗ |
| Trouble relaxing | ពិបាកក្នុងការធ្វើឲ្យបាត់ភាពតានតឹងក្នុងចិត្ត |
| Being so restless that it is hard to sit still | រសាប់រសល់ ច្រាស់ច្រាល អន្ទះអន្ទែងក្នុងខ្លួនខ្លាំងដែលពិបាកឲ្យអង្គុយស្ងៀមបាន |
| Becoming easily annoyed or irritable | ឆាប់ហ្មួហ្មង ទើសចិត្ត ឬឆាប់ខឹង |
| Feeling afraid as if something awful might happen | មានអារម្មណ៌ខ្លាច ហាក់ដូចជាមានអ្វីដែលគួរអោយខ្លាចកើតឡើង |

**Cambodian Somatic and Syndrome Inventory (C-SSI)**

| **Item​ in English** | **Item in Khmer** |
| --- | --- |
| **Over the PAST 2 WEEKS, how often have you been bothered by…​​​** | **តើញឹកញាប់កំរិតណា ដែលអ្នកត្រូវបានរំខានដោយ អាការៈខាងក្រោម៖** |
| *0 "not at all", 1 "several days", 2 "more than half the days of the 2 week period?" , 3 "nearly every day"* | *០ "មិនមានទាល់តែសោះ", ១ "ពីរបីថ្ងៃ", ២ "ច្រើនជាងមួយសប្តាហ៌",​​​​​​​​​​​​​​​​​​​​​​​​​​​​​​​​ ៣ "មានស្ទើរតែគ្រប់ពេល ឬរាល់ថ្ងៃ"* |
| ​Thinking lots | គិតច្រើន |
| ​Dizziness | វិលមុខ |
| ​Neck soreness | ​រោយក |
