# Supplementary material for: Improving recognition of common mental health disorders in Cambodia: Validation of the PHQ-9 and GAD-7 and development of a brief mental health screener
Source: PLOS Ment Health. 2025 Apr 10;2(4):e0000228. doi: 10.1371/journal.pmen.0000228 (PMC12798203; doi:10.1371/journal.pmen.0000228)
Supplement: S2 Appendix — (DOCX) [file pmen.0000228.s002.docx]

**S2 Appendix. Final 3-item screener for emotional distress in Cambodia.**

**ឧបករណ៍ត្រួតពិនិត្យសុខភាពផ្លូវចិត្ត**

| ជាង២អាទិត្យចុងក្រោយនេះ តើអ្នកត្រូវបានរំខានញឹកញាប់ យ៉ាងណាដោយសារបញ្ហា​ណាមួយដូចខាងក្រោមនេះ? សូមមេត្ដាប្រើ’’x’’ចង្អុលបង្ហាញចំលើយរបស់អ្នក | មិនមាន ទាល់តែសោះ | ពីរបីថ្ងៃ | ច្រើន ជាងមួយ សប្តាហ៍ | មានស្ទើរតែគ្រប់ ពេល ឬរាល់ថ្ងៃ |
| --- | --- | --- | --- | --- |
| 1. ពិបាកក្នុងការគេង ឬគេងលង់លក់ពិបាកងើប ឬគេងច្រើន ម៉ោងពេក | 0 | 1 | 2 | 3 |
| 1. មានអារម្មណ៍មិនល្អចំពោះខ្លួនឯង ឬមានអារម្មណ៍ថាខ្លួនមិន ជោគជ័យ  ធ្វើឲ្យខ្លួនឯង ឬគ្រួសាររបស់ខ្លួនអាប់អោនកិត្តិយស | 0 | 1 | 2 | 3 |
| 1. មានអារម្មណ៌ច្រួលច្រាលក្នុងចិត្ត អន្ទះអន្ទែង ឬតានតឹងក្នុងចិត្ត | 0 | 1 | 2 | 3 |

**ពិន្ទុសរុបទៅតាមក្រឡោន     =               _______ + _______   +** _______

**ពិន្ទុសរុប (សរុបពិន្ទុគ្រប់ក្រឡោនបញ្ចូលគ្នា)      =                       ___________________________________**

**ការដាក់ពិន្ទុ**: ប្រសិនបើពិន្ទុសរុបគឺ >/=3 នេះគឺជាលទ្ធផលវិជ្ជមាន ហើយការរៀបចំចាត់ចែងនានាគួរតែត្រូវធ្វើឡើង សម្រាប់ការបំពេញ PHQ 9 និង GAD 7 ។

**Mental Health Screener**

| **Over the last 2 weeks, how often have you been bothered by any of the following problems?**  ***(Use ”x” to indicate your answer)*** | Not at all | Several days | More than half the days | Nearly everyday |
| --- | --- | --- | --- | --- |
| 1. Trouble falling or staying asleep, or sleeping too much | 0 | 1 | 2 | 3 |
| 1. Feeling bad about yourself or that you are a failure or have let yourself or your family down | 0 | 1 | 2 | 3 |
| 1. Feeling nervous or on edge | 0 | 1 | 2 | 3 |

**Column Totals =               _______ + _______   +** _______

**TOTAL Score *(add all Column Totals)*      =  ___________________________________**

**SCORING INSTRUCTIONS**: If the Total score is  >/=3, this is a positive result and arrangements should be made for completing a PHQ 9 and GAD 7.
