# Supplementary material for: Improving recognition of common mental health disorders in Cambodia: Validation of the PHQ-9 and GAD-7 and development of a brief mental health screener
Source: PLOS Ment Health. 2025 Apr 10;2(4):e0000228. doi: 10.1371/journal.pmen.0000228 (PMC12798203; doi:10.1371/journal.pmen.0000228)
Supplement: Attachment — Submitted filename: Response to reviewers 2024-12-18.docx [file pmen.0000228.s004.docx]

**Response to Reviewers (*rebuttal is highlighted in yellow and in italics*)**

PLOS Mental Health Journal Requirements:

1. Please include a complete copy of PLOS’ questionnaire on inclusivity in global research in your revised manuscript. Our policy for research in this area aims to improve transparency in the reporting of research performed outside of researchers’ own country or community. The policy applies to researchers who have travelled to a different country to conduct research, research with Indigenous populations or their lands, and research on cultural artefacts. The questionnaire can also be requested at the journal’s discretion for any other submissions, even if these conditions are not met.  Please find more information on the policy and a link to download a blank copy of the questionnaire here: [https://journals.plos.org/mentalhealth/s/best-practices-in-research-reporting](https://urldefense.com/v3/__https:/journals.plos.org/plosmentalhealth/s/best-practices-in-research-reporting__;!!K-Hz7m0Vt54!hI4GpfSfBzOBby1w8bYDuj-GzxOq5SWAP5g_9a2b9G8q0YEmCthqsCZ-Ng6pklfwL6Dd91nv4pZSHw$). Please upload a completed version of your questionnaire as Supporting Information when you resubmit your manuscript.

*Thank you for letting us know about this form and policy to improve transparency of research that is conducted outside of researchers’ communities. We have completed the PLOS Questionnaire on Inclusivity in Global Research and uploaded it as Supporting Information.*

2. In the online submission form, you indicated that "De-identified participant data is available upon request by contacting the corresponding author.".

All PLOS journals now require all data underlying the findings described in their manuscript to be freely available to other researchers, either 
1. In a public repository, 
2. Within the manuscript itself, or 
3. Uploaded as supplementary information.

This policy applies to all data except where public deposition would breach compliance with the protocol approved by your research ethics board. If your data cannot be made publicly available for ethical or legal reasons (e.g., public availability would compromise patient privacy), please explain your reasons by return email and your exemption request will be escalated to the editor for approval. Your exemption request will be handled independently and will not hold up the peer review process but will need to be resolved should your manuscript be accepted for publication. One of the Editorial team will then be in touch if there are any issues.

*We are unable to make data freely available to researchers using a public repository, within the manuscript, or uploaded as supplementary information as this would be a breach of compliance with the UW IRB and with the Cambodian Ethics Board. We did not request to share these data when these applications were submitted initially in 2018 as this is not appropriate in Cambodia research protocols. We no longer have participant contact information were we to ask for permission to share data at this time. As such we need to remove the statement that de-identified data is available upon request. This is our first time submitting to PLOS Mental Health so apologies we were not aware of this policy until now. We are happy to discuss further.*

Additional Editor Comments (if provided):

[Note: HTML markup is below. Please do not edit.]

Reviewers' comments:

Reviewer's Responses to Questions

**Comments to the Author**

1. Does this manuscript meet PLOS Mental Health’s [publication criteria](https://urldefense.com/v3/__https:/track.editorialmanager.com/CL0/https:*2F*2Fjournals.plos.org*2Fmentalhealth*2Fs*2Fcriteria-for-publication/1/010f0192ff6d7686-8b2b658a-747a-424a-a5aa-2c5cd9041341-000000/Enf_iflM6ZndzxZ4Ls6yHVWghtTkFZobUA5Un-T1sK0=184__;JSUlJSU!!K-Hz7m0Vt54!hI4GpfSfBzOBby1w8bYDuj-GzxOq5SWAP5g_9a2b9G8q0YEmCthqsCZ-Ng6pklfwL6Dd91kqcHwPww$)? Is the manuscript technically sound, and does the data support the conclusions? The manuscript must describe methodologically and ethically rigorous research with conclusions that are appropriately drawn based on the data presented.

Reviewer #1: Yes

Reviewer #2: Partly

2. Has the statistical analysis been performed appropriately and rigorously?

Reviewer #1: I don't know

Reviewer #2: Yes

3. Have the authors made all data underlying the findings in their manuscript fully available (please refer to the Data Availability Statement at the start of the manuscript PDF file)?

The [PLOS Data policy](https://urldefense.com/v3/__https:/track.editorialmanager.com/CL0/https:*2F*2Fjournals.plos.org*2Fmentalhealth*2Fs*2Fdata-availability/1/010f0192ff6d7686-8b2b658a-747a-424a-a5aa-2c5cd9041341-000000/Rurqss8kuq3bV4Ms4ALg14F-__jx663MEyzdj1y1lLM=184__;JSUlJSU!!K-Hz7m0Vt54!hI4GpfSfBzOBby1w8bYDuj-GzxOq5SWAP5g_9a2b9G8q0YEmCthqsCZ-Ng6pklfwL6Dd91kIHs5RGQ$) requires authors to make all data underlying the findings described in their manuscript fully available without restriction, with rare exception. The data should be provided as part of the manuscript or its supporting information, or deposited in a public repository. For example, in addition to summary statistics, the data points behind means, medians and variance measures should be available. If there are restrictions on publicly sharing data—e.g. participant privacy or use of data from a third party—those must be specified.

Reviewer #1: Yes

Reviewer #2: No

4. Is the manuscript presented in an intelligible fashion and written in standard English?

PLOS Mental Health does not copyedit accepted manuscripts, so the language in submitted articles must be clear, correct, and unambiguous. Any typographical or grammatical errors should be corrected at revision, so please note any specific errors here.

Reviewer #1: No

Reviewer #2: Yes

5. Review Comments to the Author

Please use the space provided to explain your answers to the questions above. You may also include additional comments for the author, including concerns about dual publication, research ethics, or publication ethics. (Please upload your review as an attachment if it exceeds 20,000 characters)

Reviewer #1: Thank you for including the measures-perhaps this could be added as an appendix afterwards
*We have provided all of the items from the measures in Table 1 that were used in this study as a new Appendix A and the screener that was developed in this study as a new Appendix B so that the formatting is cleaner, and it is easier for PLOS Mental Health readers to utilize these tools.*

hurting yourself in some ways…,” was not included as the goal was to identify a broad screen
for distress, plus asking about suicidal thoughts within an initial assessment was considered too
invasive.-I would elaborate more on this and why perhaps phq-8 was not utilized instead

*That is correct – asking about suicidal ideation would be too invasive for a brief screener for emotional distress. However, since asking about suicide is an important first step for suicide prevention, our committee determined it was important to validate the PHQ-9 rather than the PHQ-8 to assess for accuracy in the Cambodian context. We have edited the manuscript in the Methods section on page 8 to state why the PHQ-9 was chosen over the PHQ-8. We also edited the footer for Table 2 to better complement the text on page 15 that states the suicide, and the one GAD-7 item were not included as possible screening items.*

GAD-7- which other items for multicollinearity?

*We did not include the item about being restless from the GAD-7 due to multicollinearity with the PHQ-9 8^th^ item “Or the opposite being so fidgety or restless that you have been moving around a lot more than usual.” We edited the text in the Methods section on page 15 to clarify this.*

I couldn’t read table 5

*Table 5 is the brief mental health screener that we developed in this study. We have changed it from a table to an Appendix B so that it is easier to read and more accessible to be used in future research and practice settings.*

This paper was very interesting-perhaps in future you can include 9th question in analysis.

*Thank you for this suggestion. While this is beyond the scope of the study, this data may be useful to analyze further given the limited data on suicidal ideation among Cambodians. In the meantime, we provided the prevalence of suicidal ideation from the 9^th^ item on page 17 of the manuscript in the Results section.*

Please check for grammatical errors and redundancy.

*We have reviewed the paper and made several edits to remove grammatical errors and reduce redundancy.*

Reviewer #2: This study aimed to develop a brief mental health screener applicable to the context of Cambodia, a representative low- and middle-income country (LMIC). Undoubtedly, the research holds significant public health importance, as LMICs disproportionately bear the global mental health disease burden. However, there are several major issues that need to be addressed.

1.The introduction section is lengthy regarding the mental health burden and the need for mental health services, yet it does not clearly present the importance of primary psychological screening.

*Thank you for your feedback. We have edited the Introduction on page 5 to discuss the importance of primary psychological screening to improve recognition of mental health conditions.*

2.Why did the authors choose the trauma/PSTD scale (i.e., the 16-item Harvard Trauma Questionnaire (HTQ)) as the criterion instrument to test the accuracy of the PHQ-9 and GAD-7?

*This instrument was selected given the overlap between depression, anxiety and trauma/PTSD in the Cambodian context and that the Hopkins Symptom Checklist Inventory was developed for a Western context. As such we were advised by our Cambodian research and practice colleagues to use both measures as criterion. We have revised the Methods section on page 9 to clarify why we used the HTQ in addition to the HSCL.*

3.In the methods section, the introduction of the Cambodian Somatic and Syndrome Inventory (C-SSI) appears somewhat abrupt. I recommend providing additional context to explain why the C-SSI was chosen alongside the PHQ-9 and GAD-7.

*Good point. We have edited and re-organized this paragraph on page 9 and 10 to provide more context that the C-SSI was chosen given previous research that suggests cultural idioms of distress in addition to Western constructs for depression and anxiety measured by the PHQ-9 and GAD-7 are important facets of mental health in Cambodia.*

4.It would be better to provide more details on the choice of a sample size of 500 participants and the basis for estimating that a minimum of 30% will strongly endorse symptoms.

*We have removed this sentence from page 17 as we do no longer have this detail available from when the study was designed in 2019. Other validation papers such as Udedi, 2019* [*https://bmcpsychiatry.biomedcentral.com/articles/10.1186/s12888-019-2062-2*](https://bmcpsychiatry.biomedcentral.com/articles/10.1186/s12888-019-2062-2) *(N=323), Chibanda, 2016* [*https://www.sciencedirect.com/science/article/abs/pii/S0165032715313112*](https://www.sciencedirect.com/science/article/abs/pii/S0165032715313112)*) (N=264), and in population’s with similar prevalence of depression and anxiety suggest our sample size was sufficient for this study.*

5.In the results section, why did the authors decide to omit step 7. The explanation that four items (step 4) are more feasible for a brief screening instrument for emotional distress lacks persuasiveness.

*The researchers and committee members decided to not use step 7 as a 7-item screener was deemed not feasible for use in the Cambodian health system. Our Cambodian research and practice colleagues from the start stressed the importance of a brief tool for use in under-resourced practice settings, followed by a longer assessment for people who screened positive. We have expanded the statement on page 24 about why step 7 was not used to provide this rationale.*

Furthermore, why did the authors substitute item 3 for item 4 in the Advisory Committee Review and Recommendations section?

*We have moved this section to the Discussion section on page 24 as the committee advised on the study findings. The committee suggested running the screener accuracy tests with item 3 (sleep) instead of item 4 (energy) as practitioners they felt sleep was the most common somatic complaint, they get from their Cambodian patients who are emotionally distressed. While the study was done with a sample of patients from the Diabetes Center and Mental Health Unit, the committee represented other health care settings. As such, their recommendation allows for a similarly accurate and potential more generalizable and feasible screener for use in diverse Cambodian health care contexts. We edited the section on page 24 with this detail for the reader.*

6.The study only recruited patients coming to the Cambodia-China Friendship Preah Kossamak Hospital’s Cambodia-Korea Diabetes Center and Mental Health Unit. This apparent heterogeneity in data collection methods may introduce information bias to their study results. This limitation should be acknowledged.

*Good point. We have called out this limitation in the Discussion section on page 27.*

7.The authors should explain why de-identified participant data is available only upon request, given that the PLOS Data policy requires authors to make all data underlying the findings described in their manuscript fully available without restriction, except in rare cases.

*We have reviewed our UW IRB and Cambodian Ethics Board approvals and did not obtain approval to share study data so cannot make this data fully available without restriction. Our ethics approvals were submitted five years ago when this policy was not in place, and it is not feasible for us to go back and contact study participants or revise our approvals. We had not asked for this approval initially as this public data sharing is not currently acceptable in Cambodia.*

6. PLOS authors have the option to publish the peer review history of their article ([what does this mean?](https://urldefense.com/v3/__https:/track.editorialmanager.com/CL0/https:*2F*2Fjournals.plos.org*2Fmentalthealth*2Fs*2Feditorial-and-peer-review-process*23loc-peer-review-history/1/010f0192ff6d7686-8b2b658a-747a-424a-a5aa-2c5cd9041341-000000/7afUsX-9-EyStolFOj77p45DaziXp4QJDuHc94AscgQ=184__;JSUlJSUl!!K-Hz7m0Vt54!hI4GpfSfBzOBby1w8bYDuj-GzxOq5SWAP5g_9a2b9G8q0YEmCthqsCZ-Ng6pklfwL6Dd91nz8kE02A$)). If published, this will include your full peer review and any attached files.

**Do you want your identity to be public for this peer review?** If you choose “no”, your identity will remain anonymous but your review may still be made public.


For information about this choice, including consent withdrawal, please see our [Privacy Policy](https://urldefense.com/v3/__https:/track.editorialmanager.com/CL0/https:*2F*2Fwww.plos.org*2Fprivacy-policy/1/010f0192ff6d7686-8b2b658a-747a-424a-a5aa-2c5cd9041341-000000/IZ1eYc6PlDtV1-nU5tyqg2Q5ehYL7J6X5KBecWtdlQE=184__;JSUl!!K-Hz7m0Vt54!hI4GpfSfBzOBby1w8bYDuj-GzxOq5SWAP5g_9a2b9G8q0YEmCthqsCZ-Ng6pklfwL6Dd91m3MQul0w$).

Reviewer #1: No

Reviewer #2: No

[NOTE: If reviewer comments were submitted as an attachment file, they will be attached to this email and accessible via the submission site. Please log into your account, locate the manuscript record, and check for the action link "View Attachments". If this link does not appear, there are no attachment files.]

While revising your submission, please upload your figure files to the Preflight Analysis and Conversion Engine (PACE) digital diagnostic tool, [https://pacev2.apexcovantage.com/](https://urldefense.com/v3/__https:/pacev2.apexcovantage.com/__;!!K-Hz7m0Vt54!hI4GpfSfBzOBby1w8bYDuj-GzxOq5SWAP5g_9a2b9G8q0YEmCthqsCZ-Ng6pklfwL6Dd91kouDNo6A$). PACE helps ensure that figures meet PLOS requirements. To use PACE, you must first register as a user. Registration is free. Then, login and navigate to the UPLOAD tab, where you will find detailed instructions on how to use the tool. If you encounter any issues or have any questions when using PACE, please email PLOS at figures@plos.org. Please note that Supporting Information files do not need this step.

*In compliance with data protection regulations, you may request that we remove your personal registration details at any time.* [*(Remove my information/details)*](https://urldefense.com/v3/__https:/track.editorialmanager.com/CL0/https:*2F*2Fwww.editorialmanager.com*2Fpmen*2Flogin.asp*3Fa=r/1/010f0192ff6d7686-8b2b658a-747a-424a-a5aa-2c5cd9041341-000000/b1C0Kp9m4vtVcBp4ymUVBmoMyEoyOps4d_qcojm6aFo=184__;JSUlJSU!!K-Hz7m0Vt54!hI4GpfSfBzOBby1w8bYDuj-GzxOq5SWAP5g_9a2b9G8q0YEmCthqsCZ-Ng6pklfwL6Dd91mgUdAyFA$)*. Please contact the publication office if you have any questions.*
